# Depression, anxiety and stress among healthcare workers in the context of the COVID-19 pandemic: a cross-sectional study in a tertiary hospital in Northern Vietnam

**DOI:** 10.3389/fpubh.2023.1231326

**Published:** 2023-09-19

**Authors:** Hien Thu Pham, Tung Viet Cao, Ngoc Bich Le, Nhung T-T Nguyen, Bich Thi Ngoc Vuong, Linh Vu Dieu Pham, Trang Thu Hoang, Trang Thi Hanh Pham, Thuy Ngoc Nguyen, Huong Thi Thu Bui, Tho Van Tran, Linh Thuy Vu, Phuong Thi Le

**Affiliations:** ^1^Department of Human Resources, Vietnam National Children’s Hospital, Hanoi, Vietnam; ^2^Heart Center, Vietnam National Children’s Hospital, Hanoi, Vietnam; ^3^Faculty of Fundamental Sciences, Hanoi University of Public Health, Hanoi, Vietnam; ^4^Department of Biochemistry, Vietnam National Children’s Hospital, Hanoi, Vietnam; ^5^International medical Center, Vietnam National Children’s Hospital, Hanoi, Vietnam

**Keywords:** depression, anxiety, stress, DASS 21, healthcare workers, COVID-19, Vietnam

## Abstract

**Introduction:**

The outbreak of coronavirus severe acute respiratory syndrome coronavirus 2 (SARS-CoV2) had significant effects on the mental well-being in general, particularly for healthcare professionals. This study examined the prevalence of depression, anxiety, and stress, and identified the associated risk factors amongst healthcare workers during the COVID-19 outbreak in a tertiary hospital located in Vietnam.

**Methods:**

We conducted a cross-sectional study at a tertiary-level hospital, where the Depression Anxiety and Stress Scale 21 (DASS-21) web-based questionnaire was employed. We analyzed the determinant factors by employing multivariate logistic models.

**Results:**

The prevalence of depression, anxiety, and stress symptoms were 19.2%, 24.7%, and 13.9%, respectively. Factors such as engaging in shift work during the pandemic, taking care of patients with COVID-19, and staff’s health status were associated with mental health issues among health professionals. In addition, having alternate rest periods was likely to reduce the risk of stress.

**Conclusion:**

The prevalence of mental health problems in healthcare workers during the COVID-19 pandemic was relatively high. Having resting periods could potentially mitigate the development of stress among health professionals. Our findings could be taken into account for improving mental health of the health professional population.

## 1. Introduction

The surge in the number of Coronavirus (COVID 19) cases strongly impacted public health around the world. Since the initial case in Wuhan, China, in December 2019, COVID-19 spread rapidly worldwide, quickly becoming a global health threat. As of 30 July 2023, there were over 768 million confirmed cases and over 6.9 million deaths reported globally (1). Over the same period of time, the total number of COVID-19 cases in Vietnam reached over 11.6 million confirmed cases and over 43,000 deaths (2). As a result, governments adopted a variety of measures to mitigate the spread of the virus. In Vietnam, the government enforced compulsory quarantine for people returning from abroad and patients with COVID-19; people worked from home, non-essential services were shut down, schools were suspended, there were travel restrictions, and lockdown in some locations. Such measures changed daily life and impacted incomes. Consequently, these factors affected the mental health of the population.

It is reasonable to assume that the COVID-19 pandemic was and is stressful for health workers. They had a higher risk of being infected with COVID-19 or were always fearful of being infected (3, 4). They also worked long hours, increased workloads, a shortage of personal protective equipment, faced social stigma, and lacked incentives to continue working (5–7). As a result, studies showed that this led to a significantly higher incidence of insomnia among healthcare workers as compared to non-healthcare workers during the pandemic (8, 9). However, unlike other professional groups, healthcare workers were not diagnosed and their health issues were not cared for during the pandemic. Indeed, they may not have realized that they had health problems, especially those related to mental health. This influenced the health of health care professionals and their levels of motivation. Subsequently, patient care was negatively affected.

Studies from many countries reported a prevalence of depression and anxiety in healthcare workers during the pandemic. For instance, Chen et al. (10) reported that the overall prevalence of anxiety and depression among frontline healthcare workers was 43% and 45%, respectively. Pappa et al. (11) reported that the prevalence of insomnia was 34.32% in 2020. A study in five major hospitals in Singapore and India reported that the prevalence of depression, anxiety, and stress symptoms was 10.6%, 15.7%, and 5.2%, respectively (12). A study among 1,090 medical staff in China revealed that the self-reported prevalence of anxiety symptoms, depression symptoms were 13.3%, 18.4%, and 23.9%, respectively (13). However, the percentages vary depending on the country and culture. In Vietnam, some previous studies reported the prevalence rates (14–17). For instance, Nguyen et al. reported 22.6% of participants had psychosocial problems (14). Nguyen et al. observed that 90.3% of participants felt that their job put them at risk of COVID-19 infection and 85.7% of participants expressed fear of potential infections (14). However, no study examined the associated risk factors carefully.

Vietnam experienced a challenging period of epidemic outbreaks and deployed several special strategies. For instance, mobilizing doctors from low risk countries to support high risk countries. Moreover, the healthcare staff from the studied hospital, a pediatric hospital, have worked and supported treatment on adults’ patients. These factors can contribute to an increase in the workload as well as the anxiety of healthcare workers.

The objectives of this study were to investigate the prevalence of depression, anxiety, and stress, as well as the associated risk factors, among healthcare workers at a tertiary hospital for children in Northern Vietnam during the COVID-19 outbreak. These findings will help identify strategies to support counseling services, implement stress management programs, and promote work-life balance for a particular population.

## 2. Methods

### 2.1. Study design and sample

We conducted this cross-sectional study at a tertiary-level children’s hospital in Hanoi. This is a multi-disciplinary hospital and the largest pediatric hospital in Northern Vietnam.

We recruited all permanent hospital staff in July 2022, just after the COVID-19 pandemic’s peak, to participate in the investigation. There were 1,001 staff who responded to the questionnaire (about 65% of the total hospital staff). We collected data through an online self-administered survey using an anonymous questionnaire distributed to all healthcare workers via email address. Only one response per person was permitted. The study was approved by the institutional ethics board of the Vietnam National Children’s Hospital (Number 1925/BVNTW_HĐĐĐ).

### 2.2. Measures

Depression, anxiety, and stress were assessed by the Depression Anxiety and Stress Scale 21 (DASS-21). The scale consists of three subscales that are depression, anxiety, and stress. Each subscale includes seven questions which are graded on a 4-point Likert scale from 0 to 3 (0 “Did not apply to me at all,” 1 “Applied to me to some degree, or some of the time,” 2 “Applied to me to a considerable degree, or a good part of time,” 3 “Applied to me very much, or most of the time”). The Vietnamese version of the DASS-21 scale has been translated and validated by the National Institute of Mental Health (18) with a reported Cronbach Alpha of 0.88, sufficiently reliable for the Vietnam population.

The questions also included demographic characteristics (age, gender, education, marital status, years of working, health status before COVID-19 pandemic); and working conditions during COVID-19, shift work during the pandemic, including number of working hours, having alternate rest periods, having direct contact with COVID-19 patients, incomes, number of days away from home per month, and number of sick days.

### 2.3. Data management and analysis

We extracted data and performed quality control by checking the missing values and cross-checked the information. Fortunately, we did not find duplicated records and missing records. Levels of depression, anxiety and stress were coded based on the total score as the guideline (19). For depression, total score from 0 to 9 was considered as normal, from 10 to 13 was mild, from 14 to 20 was moderate, from 21 to 27 were considered as severe, above 28 was considered extremely severe. The anxiety subscales were considered as normal (0–7), mild (8, 9), moderate (10–14), severe (15–19), and extremely severe (20–42). The total stress subscale was considered as normal (0–14), mild (15–18), moderate (19–25), severe (26–33), and extremely severe (34–42).

We employed the logistic model to investigate the associated risk factors of total depression scores equal and higher than 10, total anxiety scores equal and higher than 8, and total stress scores equal and higher than 15. Variables with *p* value above 0.2 would be included for final logistic regression models. We also conducted *t*-test and ANOVA to compare scores by participants’ characteristics, which is in the appendix. The significant level was set at 0.05.

## 3. Results

The mean age (standard deviation—SD) was 35.7 (± 13.5) years. More than 75% of participants were female and most participants were married (84%). Approximately 23.9% were doctors and 56.2% were nurses or medical technologists. More than half of the study participants had more than 10 years of experience in their respective fields (Table 1).

**Table 1:** Participants’ characteristics of an children’s hospital, location in Northern Vietnam all and by type of professional, 2022.

Characteristics	Total *n* = 1,001	Doctors *n* = 239	Nurses/medical technologists *n* = 563	Other staff *n* = 199
	n (%)	n (%)	n (%)	n (%)
Age (Mean: 35.7; SD: 13.5)				
≤35	502 (50.1)	102 (42.7)	315 (56.0)	85 (42.7)
>35	499 (49.9)	137 (57.3)	248 (44.0)	114 (57.3)
Gender				
Male	245 (24.5)	108 (45.2)	86 (15.3)	51 (25.6)
Female	756 (75.5)	131 (54.8)	477 (84.7)	148 (74.4)
Education				
Junior college or below	238 (23.8)	0 (0.0)	171 (30.4)	67 (33.7)
Bachelor’s degree	410 (41.0)	27 (11.3)	291 (51.7)	92 (46.2)
Master degree or above	353 (35.3)	212 (88.7)	101 (17.9)	40 (20.1)
Marital status				
Married	841 (84.0)	204 (85.4)	477 (84.7)	160 (80.4)
Single	160 (16.0)	35 (14.6)	86 (15.3)	39 (19.6)
Working years				
<5 years	210 (21.0)	64 (26.8)	97 (17.2)	49 (24.6)
5–10 years	220 (22.0)	46 (19.2)	134 (23.8)	40 (20.1)
>10 years	571 (57.0)	129 (54.0)	332 (59.0)	110 (55.3)
Health status before COVID-19				
Very good/good	805 (80.4)	194 (81.2)	453 (80.5)	158 (79.4)
Weak	196 (19.6)	45 (18.8)	110 (19.5)	41 (20.6)
Working hours				
Regular work hours (8 h/day)	679 (70.2)	198 (86.5)	324 (58.9)	157 (83.5)
Shiftwork	288 (29.8)	31 (13.5)	226 (41.1)	31 (16.5)
Direct contact with COVID-19 patients				
No	237 (24.5)	43 (18.8)	110 (20.0)	84 (44.7)
Yes	730 (75.5)	186 (81.2)	440 (80.0)	104 (55.3)
Having alternate rest period				
No	130 (13.4)	42 (18.3)	63 (11.5)	25 (13.3)
Yes	837 (86.6)	187 (81.7)	487 (88.5)	163 (86.7)
Income				
<10 million VND	646 (64.5)	120 (50.2)	369 (65.5)	157 (78.9)
10–20 million VND	305 (30.5)	87 (36.4)	182 (32.3)	36 (18.1)
>20 million VND	50 (5.0)	32 (13.4)	12 (2.1)	6 (3.0)
Number of days away from home/month				
None	117 (12.1)	16 (7.0)	46 (8.4)	55 (29.3)
<10 days	608 (62.9)	168 (73.4)	343 (62.4)	97 (51.6)
10–30 days	174 (18.0)	30 (13.1)	112 (20.4)	32 (17.0)
>30 days	68 (7.0)	15 (6.6)	49 (8.9)	4 (2.1)
Number of sick days				
None	335 (34.6)	83 (36.2)	193 (35.1)	59 (31.4)
<10 days	532 (55.0)	124 (54.1)	298 (54.2)	110 (58.5)
10–30 days	93 (9.6)	20 (8.7)	54 (9.8)	19 (10.1)
>30 days	7 (0.7)	2 (0.9)	5 (0.9)	0 (0.0)

In relation to working conditions of healthcare workers during the COVID-19 pandemic, about 70.2% of respondents had normal work hours (8 h per day) and 29.8% were shift workers. Most participants (86.6%) had alternate rests. About 75.5% of the staff had direct contact with COVID-19 patients. On average, 64.5% had income less than 10 million VND, and 30.5% had income from 10 to 20 million VND.

Table 2 shows the percentage of depression, anxiety, and stress among healthcare workers. 24.7% of respondents reported having symptoms of anxiety, of which 11.7% had moderate symptoms and 5.4% had extremely severe symptoms. 19.2% of respondents reported having symptoms of depression, of which 8.3% had mild symptoms and 6.5% had mild symptoms. 13.9% of respondents reported having symptoms of stress, of which 5.4% had mild symptoms and 3.8% had moderate symptoms.

**Table 2:** Levels of depression, anxiety and stress symptoms among the staff in a children hospital located in the Northen Vietnam, in total and by professionals.

Characteristics	Total *n* = 1,001	Doctors *n* = 239	Nurses/medical technologists *n* = 563	Other staff *n* = 199
	n (%)	n (%)	n (%)	n (%)
Depression				
Normal	809 (80.8)	198 (82.8)	446 (79.2)	165 (82.9)
Mild	65 (6.5)	12 (5.0)	42 (7.5)	11 (5.5)
Moderate	83 (8.3)	18 (7.5)	48 (8.5)	17 (8.5)
Severe	11 (1.1)	2 (0.8)	8 (1.4)	1 (0.5)
Extremely severe	33 (3.3)	9 (3.8)	19 (3.4)	5 (2.5)
Anxiety				
Normal	754 (75.3)	198 (82.8)	400 (71.0)	156 (78.4)
Mild	45 (4.5)	7 (2.9)	29 (5.2)	9 (4.5)
Moderate	117 (11.7)	17 (7.1)	82 (14.6)	18 (9.0)
Severe	31 (3.1)	5 (2.1)	19 (3.4)	7 (3.5)
Extremely severe	54 (5.4)	12 (5.0)	33 (5.9)	9 (4.5)
Stress				
Normal	862 (86.1)	204 (85.4)	479 (85.1)	179 (89.9)
Mild	54 (5.4)	14 (5.9)	35 (6.2)	5 (2.5)
Moderate	38 (3.8)	9 (3.8)	23 (4.1)	6 (3.0)
Severe	31 (3.1)	7 (2.9)	19 (3.4)	5 (2.5)
Extremely severe	16 (1.6)	5 (2.1)	7 (1.2)	4 (2.0)

The associated factors of depression, anxiety, and stress are presented in Tables 3–5, respectively. Sociodemographic characteristics (i.e., age, gender, education, marital status, year of experience) were not associated with symptoms of depression, anxiety, and stress. The odds of having depression were significantly higher among those having weak health status before the outbreak of COVID-19 (OR = 1.87, 95% CI 1.28–2.73). Similarly, high proportions of those suffering from anxiety among health professionals were those with shift work during the pandemic (OR = 1.48, 95% CI 1.06–2.07) and having weak health status before the outbreak of COVID-19 (OR = 1.67, 95% CI 1.16–2.40). High risk stress was observed in those in direct contact with COVID-19 patients (OR = 1.94, 95%CI 1.13–3.32) and shift work during the pandemic (OR = 2.22, 95%CI 1.47–3.37). Healthcare workers having alternate rest periods significantly decreased the odds of having stress (OR = 0.42, 95%CI 0.26–0.67).

**Table 3:** Associations between participants’ characteristics and depression’s prevalence in a children hospital located in Northern Vietnam, 2022.

	Depression score ≥ 10	Depression score ≤ 9	OR	95% CI of OR	*P*
	n (%)	n (%)			
Education					
Junior college or below	44 (18.4)	194 (81.5)	1	–	–
Bachelor’s degree	90 (21.9)	320 (78.0)	1.05	0.65–1.68	0.850
Master degree or above	58 (16.4)	295 (83.5)	0.89	0.59–1.32	0.552
Health status before COVID-19					
Very good/good	138 (17.1)	667 (82.9)	1	–	–
Weak	**54 (27.6)**	**142 (72.4)**	**1.87**	**1.28–2.73**	**0.001**
Working hours					
Normal work hours (8 h/day)	118 (17.4)	561 (82.6)	1	–	–
Shiftwork	72 (25.0)	216 (75.0)	1.43	1–2.06	0.049
Direct contact with COVID-19 patients					
No	37 (15.6)	200 (84.4)	1	–	–
Yes	153 (21.0)	577 (79.0)	1.40	0.93–2.13	0.109
Having alternate rest					
No	33 (25.4)	97 (74.6)	1	–	–
Yes	157 (18.8)	680 (81.2)	0.71	0.45–1.11	0.133
Income					
<10 million VND	144 (22.3)	502 (77.7)	1	–	–
10–20 million VND	42 (13.8)	263 (86.2)	0.49	0.2–1.23	0.127
>20 million VND	6 (12.0)	44 (88.0)	0.93	0.36–2.39	0.883
Number of days away from home/month					
None	25 (21.4)	92 (78.6)	1	–	–
<10 days	106 (17.4)	502 (82.6)	0.93	0.43–2	0.856
10–30 days	43 (24.7)	131 (75.3)	1.29	0.69–2.41	0.428
>30 days	16 (23.5)	52 (76.5)	0.97	0.49–1.91	0.920

Bold values highlight statistically significant association with *p* < 0.05.

**Table 4:** Associations between participants’ characteristics and anxiety’s prevalence in a children hospital located in Northern Vietnam, 2022.

	Anxiety score ≥ 8	Anxiety score ≤ 7	OR	95% CI of OR	*p*
	n (%)	n (%)			
Education					
Junior college or below	63 (26.5)	175 (73.5)	1	–	–
Bachelor’s degree	120 (29.3)	290 (70.7)	0.86	0.52–1.43	0.558
Master degree or above	64 (18.1)	289 (81.9)	0.76	0.49–1.18	0.219
Marital status					
Married	212 (25.2)	629 (74.8)	1	–	–
Unmarried/Divorced/widowed	35 (21.9)	125 (78.1)	0.77	0.5–1.2	0.257
Professional position					
Doctors	41 (17.2)	198 (82.8)	1	–	–
Nurses/medical technologists	163 (29.0)	400 (71.0)	0.90	0.5–1.62	0.726
Other staff	43 (21.6)	156 (78.4)	0.68	0.44–1.04	0.074
Health status before COVID-19					
Very good/good	181 (22.5)	624 (77.5)	1	–	–
Weak	**66 (33.7)**	**130 (66.3)**	**1.67**	**1.16–2.4**	**0.006**
Working hours					
Normal work hours (8 h/day)	144 (21.2)	535 (78.8)	1	–	–
Shiftwork	**97 (33.7)**	**191 (66.3)**	**1.48**	**1.06–2.07**	**0.023**
Having alternate rest					
No	36 (27.7)	94 (72.3)	1	–	–
Yes	205 (24.5)	632 (75.5)	0.86	0.55–1.33	0.502
Income					
<10 million VND	181 (28.1)	465 (71.9)	1	–	–
10–20 million VND	**62 (20.3)**	**243 (79.7)**	**0.27**	**0.09–0.8**	**0.018**
>20 million VND	4 (8.0)	46 (92.0)	0.46	0.16–1.37	0.163
Number of days away from home/month					
None	29 (24.8)	88 (75.2)	1	–	–
<10 days	133 (21.9)	475 (78.1)	1.30	0.64–2.64	0.473
10–30 days	57 (32.8)	117 (67.2)	1.60	0.9–2.85	0.110
>30 days	22 (32.4)	46 (67.6)	1.08	0.57–2.02	0.818

Bold values highlight statistically significant association with *p* < 0.05.

**Table 5:** Associations between participants’ characteristics and Stress’s prevalence in a children hospital located in Northern Vietnam, 2022.

	Stress score ≥ 15	Stress score ≤ 14	OR	95% CI of OR	*P*
	n (%)	n (%)			
Education					
Junior college or below (reference)	24 (10.1)	214 (89.9)	1	–	–
Bachelor’s degree	71 (17.3)	339 (82.7)	1.37	0.7–2.7	0.362
Master degree or above	44 (12.5)	309 (87.5)	0.69	0.4–1.18	0.176
Professional position					
Doctors (reference)	35 (14.6)	204 (85.4)	1	–	–
Nurses/medical technologists	**84 (14.9)**	**479 (85.1)**	**0.48**	**0.23–0.99**	**0.046**
Other staff	20 (10.1)	179 (89.9)	0.64	0.36–1.14	0.131
Health status before COVID-19					
Very good/good (reference)	104 (12.9)	701 (87.1)	1	–	–
Weak	35 (17.9)	161 (82.1)	1.31	0.83–2.05	0.242
Working hours					
Normal work hours (8 h/day) (reference)	73 (10.8)	606 (89.2)			
Shiftwork	**62 (21.5)**	**226 (78.5)**	**2.22**	**1.47–3.37**	**0.000**
Direct contact with COVID-19 patients					
No (reference)	21 (8.9)	216 (91.1)	1	–	–
Yes	**114 (15.6)**	**616 (84.4)**	**1.94**	**1.13–3.32**	**0.015**
Having alternate rest					
No (reference)	34 (26.2)	96 (73.8)	1	–	–
Yes	**101 (12.1)**	**736 (87.9)**	**0.42**	**0.26–0.67**	**0.000**
Income					
<10 million VND (reference)	103 (15.9)	543 (84.1)	1	–	–
10–20 million VND	32 (10.5)	273 (89.5)	0.46	0.16–1.39	0.170
>20 million VND	4 (8.0)	46 (92.0)	0.85	0.27–2.61	0.771
Number of days away from home/month					
None (reference)	18 (15.4)	99 (84.6)	1	–	–
<10 days	76 (12.5)	532 (87.5)	0.50	0.2–1.27	0.147
10–30 days	32 (18.4)	142 (81.6)	0.75	0.35–1.63	0.471
>30 days	9 (13.2)	59 (86.8)	0.59	0.26–1.37	0.220

Bold values highlight statistically significant association with *p* < 0.05.

## 4. Discussion

Mental health problems among healthcare worker can lead to high levels of job dissatisfaction and increased turnover (20). So, the evidence of the mental health problems concern can help address the issue by creating a supportive work environment and promoting staff retention. Consequently this contributes to better continuity of care for patients and hospital performance (21). In this study, the prevalence of depression, anxiety and stress among healthcare workers were 19.2%, 24.7%, and 13.9%, respectively. Those were slightly higher than figures reported by some other studies in Vietnam (16, 17). However, figures in our study were lower than those in a study conducted during the fourth wave of COVID-19 (22). The prevalence of depression, anxiety, and stress among healthcare workers in this study was lower than figures in many countries, including China (23.6%, 27.4%, and 16.3% respectively) (23), South Korea (30.6%, 41%, and 19.4% respectively) (24), Italy (35.9%, 25.5%, 33.3%, respectively) (25), Brazil (38.4%, 53.8%, and 40.3%, respectively) (26), and Northwest Ethiopia (55.3%, 69.6%, and 20.5%, respectively) (27). The prevalence of depression, anxiety, and stress among healthcare workers in this study was higher than figures in Singapore and India (12). Nonetheless, in line with the other countries, these prevalence rates in health providers in Vietnam during COVID-19 were higher than in the larger community.

During the pandemic, health professionals experienced many psychosocial stressors such as the disruption of routine life, travel restrictions, shortage of necessities, separation from family members and friends, and salary reduction. Indeed, during the COVID-19 pandemic, several frontline healthcare workers such as doctors working with COVID-19 patients or laboratories, were isolated with other staff in the hospital and isolated with families and communities due to incomplete information and fear associated with COVID-19 (16). In the beginning of pandemic, Holmes et al. (28) had called for actions to address the mental health in vulnerable groups including healthcare providers and emphasized the long term psychological impact. In this study, even at the end of the peak wave of pandemic when life was gradually returning to normal, the prevalence remained high. Therefore, further studies on mental health issues in health workers need to be conducted in order to promote the healthcare sector.

The lower prevalence of depression, anxiety, and stress observed among healthcare workers in our study may be attributed to several factors. Firstly, our study was conducted once the pandemic had ended and life had returned to a more normal state. During this time, there was a clearer understanding of virus transmission and spread, which likely reduced the fear and uncertainty experienced by healthcare workers. As mentioned by Singh and Subedi (29) health workers initially faced fear, threats, and eviction from their homes due to concerns about bringing the virus home in the beginning stage of pandemic. Such experiences often led to stigma, discrimination, and social isolation. Secondly, Vietnam adopted several effective strategies to control COVID-19 in the last stage (30). These strategies likely contributed to a lower number of cases and reduced the burden on healthcare workers, resulting in less psychological distress. Thirdly, the staff studied were in a children’s hospital where the number of COVID-19 hospitalized cases was small. Some staff had to mobilize to support other hospitals, but this was a small number. During the outbreak in Ho Chi Minh City and the southern provinces, 195 healthcare workers of a studied hospital traveled south to help deal with the pandemic. Healthcare workers at the hospital took alternate breaks from 7 to 14 days per month. Incomes were cut based on the number of actual working days. Healthcare workers with COVID-19 infections are entitled to 7–10 days of paid quarantine leave according to the social insurance regime.

In conclusion, our study supported the fact that mental health depends on the healthcare workers’ emotional response under pressurized situations (31) or adaptation to contextual demands (32). We suggest using the context sensitivity index (CSI) to measuring the ability to identify the presence and absence of stressor context cues in Vietnam.

Our study revealed that those with shiftwork were more likely to suffer from anxiety and stress than those with regular work hours (8 h/day). A study from Korea showed that female nurses or nursing assistants who did shift work had a higher risk of anxiety (33). Another study revealed that nurses working night shifts were at twice the risk of developing stress than those working the day shifts (27). In hospitals, clinical staff members often do shiftwork, whereas the administrative staff work regular hours. Hence we observed a higher prevalence of stress amongst the healthcare workers working directly with patients.

Healthcare workers in direct contact with patients with COVID-19 were more likely to have stress. Previous findings from other countries also showed that healthcare workers in direct exposure to patients with COVID-19 were at a higher risk of mental health problems (34–37). A study conducted in the fourth wave of COVID-19 in Vietnam reported that healthcare workers treating moderate and severe COVID-19 patients were at increased risk for anxiety (22).

Our study also found that those having alternate rest periods significantly decreased the risk of having stress. A study conducted by Robles et al. with 5,938 healthcare workers found that over 30% of frontline healthcare workers reported a lack of rest time, and those with a lack of rest time were at a 3.1 times higher risk of having insomnia (38). Several qualitative studies revealed that healthcare workers desired adequate rest during COVID-19. They would like more support and attention toward their psychological well-being from leaders (39, 40). We also suggest special implement intervention for clinical staff in Vietnam.

Some intervention programs to cope with new psychosocial issues resulting from COVID-19 for healthcare workers have been introduced. For example, the Institute of Mental Health and the Medical Psychology Research Center of the Second Xiangya Hospital provided psychological support by examining of immediate needs from the staff and adjusting the measures afterward (20). In this study, the hospital provided a place for rest, protective supplies, and training on psychological skills to deal with patients’ emotional problems such as anxiety or depression during the pandemic. The trial entitled “RECHARGE” mainly focuses on psychoeducation by teaching people techniques on problem-solving skills and managing worries in Australia, Switzerland (41). A program in Canada had been using a Virtual Peer Support Platform to guide healthcare workers to build resilience against burnout by group therapy (42). Regardless of the methodology, all studies emphasized the significance of multidisciplinary collaboration. However, most past programs were implemented in university associated hospitals, which prevents us from applying the findings to other types of hospital or “lack a rigorous protocol” impedes finding out the best way to go (21). Therefore, the World Health Organization is still calling to develop a tailored psychological intervention for healthcare workers worldwide (43).

Though DASS-21 has been widely used to assess levels of depression, anxiety, and stress in various research, it has certain limitations that can lead to subscale overlap and interaction. In the current study, we found strong positive correlations between depression and anxiety (*r* = 0.85, *p* < 0.05), depression and stress (*r* = 0.88, *p* < 0.05), and anxiety and stress (*r* = 0.84, *p* < 0.05). Indeed, we observed the consistent determinant factors for each subscale (Tables 3–5). The reasons might be attributed to the scale reliance on self-reporting, in other words, it is influenced by individual socially desirable response. Furthermore, there is the potential of bias due to cultural factors in the questionnaire (44, 45). Therefore, findings from this study could be considered as preliminary results. Future comprehensive studies should combine its findings with other assessment methods, taking account of the cultural context. This can help mitigate some limitations of the scale.

## 5. Limitations

This study is subject to several limitations. Firstly, the research design was a cross-sectional study, so causal relationships are inconclusive. Secondly, given that the study took place after the pandemic, there could have been recall bias involved when obtaining information.

Finally, it is important to note that the study was conducted at a single center, which may limit the scope of the findings. Nonetheless, our sample size is high (above 1,000), hence, the interpreting findings are reliable.

## 6. Conclusion

The prevalence of depression, anxiety, and stress among healthcare workers were notably high. Additionally, staff with adverse working conditions, such as shift work, direct contact with COVID-19 patients, and income level, and psychological status before the pandemic, were more likely to have a high risk of mental health problems. Having alternate rest periods and limiting time away from home to no more than 10 days per month during COVID-19 pandemic might reduce risk of stress development. The findings of the study can help promote adequate measures to protect the mental health of pediatric health staff during pandemics.

## Data availability statement

The original contributions presented in the study are included in the article/Supplementary material, further inquiries can be directed to the corresponding author.

## Ethics statement

The studies involving humans were approved by the Institutional Review Board at the Vietnam National Children’s Hospital (IRB number:1925/BVNTW-HDDD dated August 22, 2022). The studies were conducted in accordance with the local legislation and institutional requirements. The participants provided their written informed consent to participate in this study.

## Author contributions

HTP: conceptualization, methodology, and writing—review and editing. TV: conceptualization, methodology, and review and editing. NT-T and NB: writing—original draft and writing—review and editing. LV, BT, LT, TV, TT, PT, TTH, HTTB, and TN: data curation, formal analysis and writing - original draft. All authors contributed to the article and approved the submitted version.

## Conflict of interest

The authors declare that the research was conducted in the absence of any commercial or financial relationships that could be construed as a potential conflict of interest.

## Publisher’s note

All claims expressed in this article are solely those of the authors and do not necessarily represent those of their affiliated organizations, or those of the publisher, the editors and the reviewers. Any product that may be evaluated in this article, or claim that may be made by its manufacturer, is not guaranteed or endorsed by the publisher.
