# Supplementary material for: Depression, anxiety and stress among healthcare workers in the context of the COVID-19 pandemic: a cross-sectional study in a tertiary hospital in Northern Vietnam
Source: Front Public Health. 2023 Sep 19;11:1231326. doi: 10.3389/fpubh.2023.1231326 (PMC10546943; doi:10.3389/fpubh.2023.1231326)
Supplement: Supplementary file 1 [file Data_Sheet_1.PDF]

Appendix 1: Associations between participants' characteristics and depression score, anxiety score and stress score

Participant's Characteristics	Depression Score		Anxiety Score		Stress Score	
	Mean ±SD	p	Mean ±SD	p	Mean ±SD	p
**Age**						
≤ 35	4.8 ± 7.40	0.292	5.1 ± 6.97	0.323	7.3 ± 8.07	0.225
> 35	4.3 ± 7.58		4.7 ± 7.66		6.6 ± 8.44	
**Gender**						
Male	4.7 ± 7.94	0.746	4.4 ± 7.53	0.253	7.2 ± 9.11	0.653
Female	4.5 ± 7.35		5 ± 7.25		6.9 ± 7.97	
**Education**						
Junior college or below	3.8 ± 6.41	0.025	5 ± 6.81	<0.001	6.3 ± 7.43	0.012
Bachelor's degree	5.3 ± 8.48		5.8 ± 8.13		7.9 ± 8.92	
Master degree or above	4.1 ± 6.86		3.7 ± 6.46		6.3 ± 7.9	
**Marital status**						
Married	4.5 ± 7.52	0.550	5.0 ± 7.57	0.152	6.9 ± 8.32	0.834
Unmarried/Divorced/widowed	4.9 ± 7.36		4.3 ± 5.82		7.1 ± 7.97	
**Professional position**						
Doctors	4.4 ± 7.53	0.298	3.7 ± 6.76	0.001	6.6 ± 8.5	0.083
Nurses/ medical technologists	4.8 ± 7.56		5.6 ± 7.63		7.4 ± 8.2	
Other staffs	3.9 ± 7.25		4.3 ± 6.83		6 ± 8.08	
**Working years**						
< 5 years	4.6 ± 7.27	0.735	4.5 ± 6.3	0.484	6.8 ± 7.82	0.870
5 – 10 years	4.8 ± 7.31		5.3 ± 7.1		7.2 ± 8.07	
> 10 years	4.4 ± 7.65		4.9 ± 7.74		6.9 ± 8.5	
**Health status before COVID-19**						
Very good/good	4.1 ± 7.1	0.002	4.6 ± 7.12	0.008	6.5 ± 7.98	0.002
Normal/ weak	6.2 ± 8.74		6.2 ± 7.97		8.7 ± 9.12	
**Working hours**						
Normal work hours (8h/day)	4.1 ± 6.87	0.003	4.1 ± 6.55	<0.001	6.3 ± 7.69	<0.001
Shiftwork	5.8 ± 8.9		6.9 ± 8.7		8.6 ± 9.45	
**Direct contact with COVID-19 patients**						
No	4.0 ± 7.11	0.169	4.4 ± 6.83	0.210	6.5 ± 8.18	0.239
Yes	4.8 ± 7.71		5.1 ± 7.53		7.2 ± 8.36	
**Having alternate rest**						
No	5.8 ± 8.15	0.067	5.7 ± 7.62	0.184	8.8 ± 9.31	0.014
Yes	4.4 ± 7.46		4.8 ± 7.32		6.7 ± 8.12	
**Income**						

< 10 million VND	5.1 ± 8.03	0.001	5.5 ± 7.81	<0.001	7.6 ± 8.77	0.002
10 – 20 million VND	3.5 ± 6.37		4 ± 6.33		6.0 ± 7.2	
> 20 million VND	2.7 ± 5.59		2 ± 4.88		4.3 ± 6.42	
**Number of days away from home/month**						
None	4.1 ± 6.93	0.003	4.7 ± 6.66	<0.001	6.4 ± 8.22	0.006
< 10 days	4.1 ± 6.88		4.2 ± 6.58		6.5 ± 7.66	
10 – 30 days	6.4 ± 9.8		7 ± 9.62		8.8 ± 10.02	
> 30 days	5.3 ± 7.39		6 ± 7.57		8.2 ± 8.78	
**Number of sick days**						
None	4.1 ± 7.04	0.410	4.5 ± 7	0.510	6.6 ± 8.12	0.597
< 10 days	4.9 ± 8.03		5.2 ± 7.72		7.1 ± 8.56	
10 – 30 days	4.3 ± 6.78		4.8 ± 6.67		7.4 ± 7.79	
> 30 days	6.5 ± 4.85		6 ± 5.77		9.4 ± 6.29	
